# Metformin use in gestational diabetes mellitus and neonatal outcomes: a systematic review and meta-analysis on the risk of small for gestational age

**DOI:** 10.3389/fmed.2025.1737337

**Published:** 2026-01-15

**Authors:** Wenwen Zhang, Liwei Ren, Juan Du, Ruijia Sun, Wenqi Zhao, Xueqing Song, Shuo Jiang, Zhao Wang, Wenjuan Wang

**Affiliations:** 1 School of Clinical and Basic Medical Sciences, Shandong First Medical University & Shandong Academy of Medical Sciences, Jinan, China; 2 Department of Pediatrics, Central Hospital Affiliated to Shandong First Medical University, Jinan, China; 3 School of Laboratory Animal & Shandong Laboratory Animal Center, Shandong First Medical University & Shandong Academy of Medical Sciences, Jinan, China

**Keywords:** gestational diabetes mellitus (GDM), insulin, meta-analysis, metformin, small for gestational age (SGA)

## Abstract

**Purpose:**

It remains unclear whether maternal metformin use in gestational diabetes mellitus (GDM) is associated with an increased risk of small-for-gestational-age (SGA) newborns.

**Methods and results:**

A systematic literature search was conducted across PubMed, Embase, and the Cochrane Library up to September 1, 2024. Nineteen studies (*n* = 115,192 participants), comprising randomized controlled trials and cohort studies, were included. Two evaluators independently assessed eligibility and bias risk. Maternal use of metformin for GDM was not significantly associated with SGA incidence in newborns (OR = 1.10, 95% CI: 0.97–1.24, *p* = 0.14). No notable differences were observed compared to insulin-treated (*n* = 27,622, OR = 1.25, 95% CI: 0.91–1.73, *p* = 0.17) or placebo groups (*n* = 1,685, OR = 1.36, 95% CI: 0.80–2.32, *p* = 0.26). However, two studies (*n* = 554) indicated a lower SGA incidence with metformin than with diet modification therapy (OR = 0.50, 95% CI: 0.29–0.87, *p* = 0.01).

**Conclusions:**

Maternal metformin use in the management of GDM doesn't increase SGA risk in offspring, suggesting its relative safety and effectiveness in this context. Further research is required to explore metformin's long-term effects of metformin on offspring of mothers with GDM.

**Systematic Review Registration:**

https://www.crd.york.ac.uk/PROSPERO/view/CRD420251141984, identifier CRD420251141984.

## Introduction

Gestational diabetes mellitus (GDM), defined as glucose intolerance with onset or first recognition during pregnancy, is now recognized as one of the most prevalent pregnancy-related complications worldwide (1, 2). GDM can increase the risk of perinatal complications and adversely affect long-term metabolic health in both mothers and their offspring. Metabolic diseases pose a serious threat to human health and have become a major global public health issue (3). Therefore, effective management of blood glucose levels in mothers with GDM is essential to prevent intergenerational transmission of metabolic diseases. There are a variety of clinical treatments for GDM, including dietary modifications, increased physical activities, oral hypoglycemic drugs, and other pharmacological therapies (4, 5). Among these, metformin is widely used due to its efficacy, safety, and low cost. As a first-line therapy, metformin has many advantages, including improvements in hemoglobin A1C (HbA1c), weight, and cardiovascular outcomes. Unless contraindicated, metformin is considered an initial pharmacological option for managing diabetes (6, 7). With the rising prevalence of GDM, the use of metformin during pregnancy has become increasingly common. However, recent evidence suggests that metformin crosses the placenta through passive diffusion, potentially reaching fetal concentrations equal to or exceeding maternal levels, thereby leading to concerns about potential effects on fetal and placental growth (8, 9). Animal studies have indicated that fetal accumulation of metformin is linked to growth restriction, as seen the emergence of SGA in the offspring of primates exposed to the drug within 30 days after conception (10). In human studies, the association between maternal metformin use and small-for-gestational-age (SGA) infants, defined as birth weight below the 10th percentile for gestational age, remains poorly characterized. Current evidence is conflicting, with some studies suggesting an increased risk of SGA (11), while others report no significant association (10). This inconsistency may stem from heterogeneity across study populations, diagnostic criteria, and treatment protocols. Furthermore, the mechanisms underlying potential effects of metformin on fetal growth require further elucidation. Given these uncertainties, it is necessary to systematically evaluate the evidence regarding the effects of metformin on fetal growth outcomes. Therefore, we conducted a meta-analysis to assess the association between maternal metformin use during pregnancy and SGA risk in offspring. The findings of this study will provide evidence-based recommendations to help optimize clinical decision-making in the management of GDM and reduce adverse neonatal outcomes.

## Methods

### Literature searches, search strategies, and eligibility

A systematic literature search was performed using predefined search terms in PubMed (June 1997 to September 1, 2024), Embase (1974 to September 1, 2024), and the Cochrane Library (from inception to September 1, 2024). No filters were applied, language or location restrictions were applied.

In the included studies, the diagnosis of GDM was based on the diagnostic criteria of the respective local medical centers where each study was conducted. We did not exclude literature based on differences in GDM diagnostic criteria. Conference abstracts were eligible only if they provided sufficient data for evaluation, no abstracts met this criterion and thus were excluded.

### Document screening and data extraction

**Figure 1:**
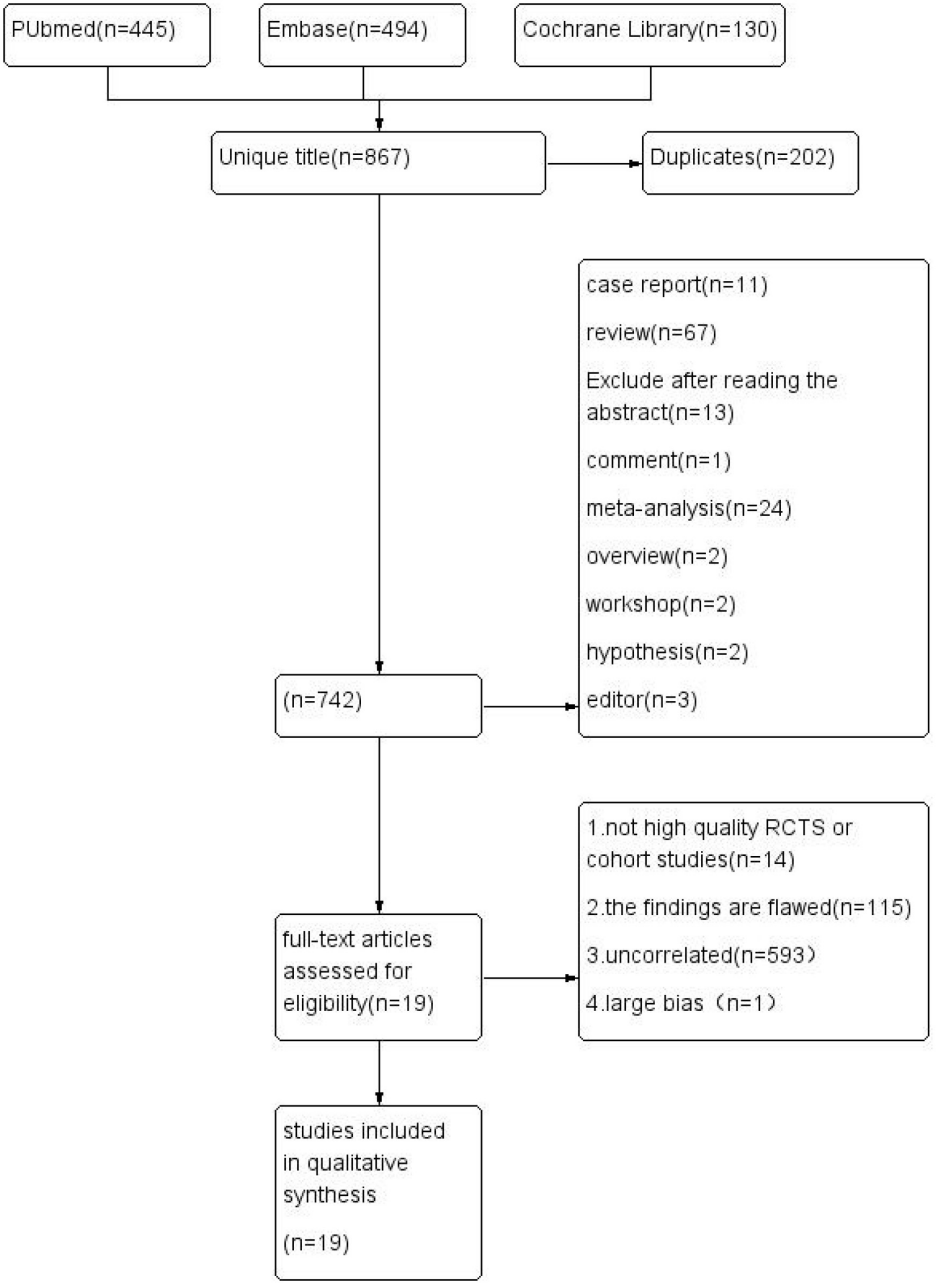
Flow diagram of studies identified in the systematic review. This flowchart summarizes the process of evidence search and study selection for this meta-analysis, including the inclusion and exclusion criteria applied at each stage. The analysis focuses on the relationship between metformin use and small for gestational age (SGA) (defined as birth weight below the 10th percentile for gestational age).

**Figure 2:**
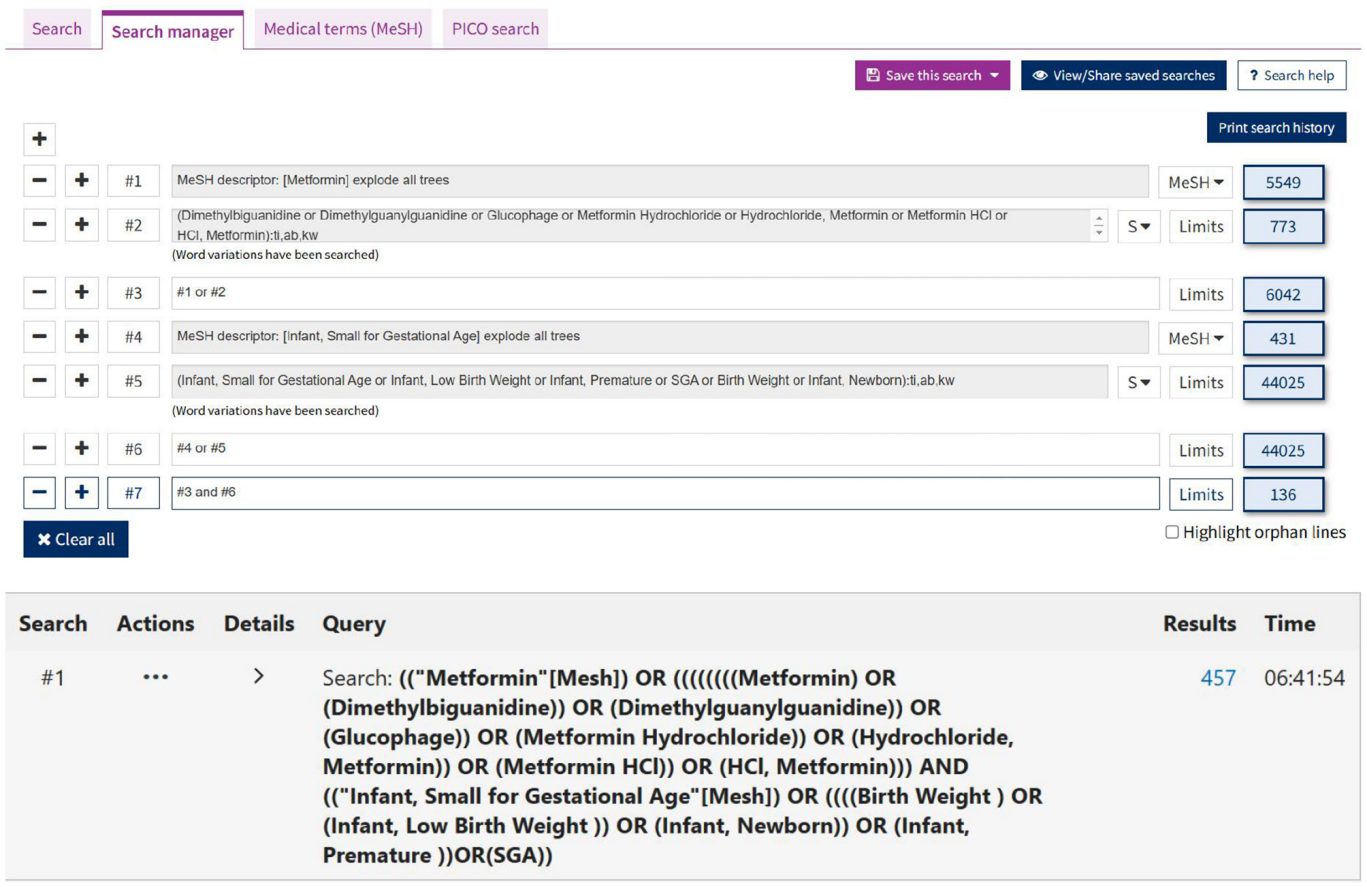
Screenshot of the search interface displaying keywords and databases used (PubMed, Embase, Cochrane Library) in the systematic search for studies on metformin, GDM, and SGA.

Data extraction and quality assessment were conducted independently by two reviewers. Disagreements were resolved by consensus or by consulting a third reviewer. The search terms included: Metformin-related terms: (1) Metformin OR Dimethylbiguanidine OR Dimethylguanylguanidine OR Glucophage; (2) Hydrochloride OR Hydrochloride, Metformin OR Metformin HCl OR HCl, Metformin. Infant-related terms: Infant, Small for Gestational Age OR Infant, Low Birth Weight OR Infant, Premature OR SGA; (3) Birth-related terms: Birth Weight OR Infant, Newborn. An initial screening of titles and abstracts was conducted, followed by a comprehensive review of full texts. The results of each stage of the review process were summarized in the flowchart (Figure 1). Additionally, details of the search strategy details were shown in Figure 2.

### Risk of bias assessment

**Figure 3:**
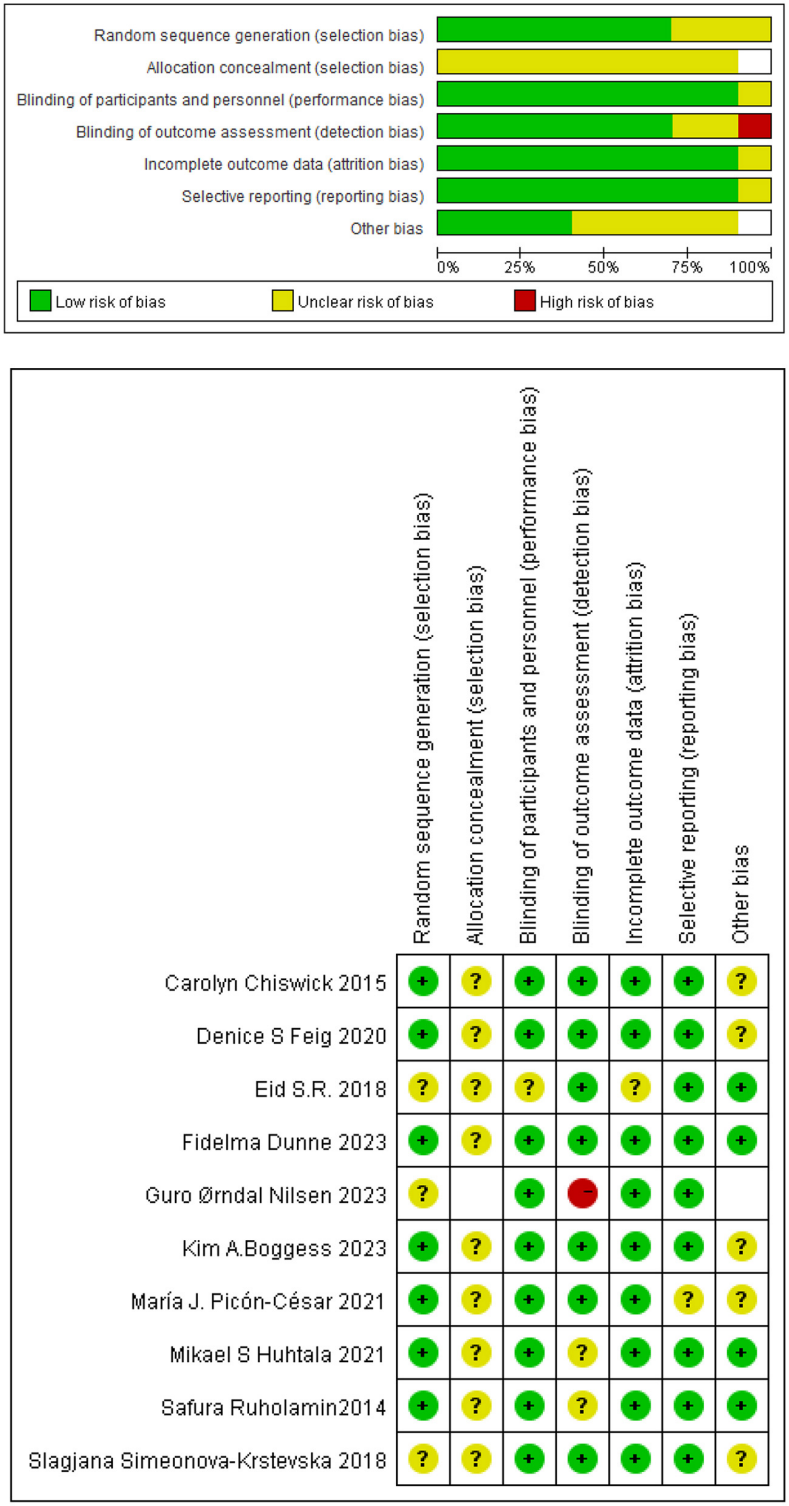
Risk of bias summary in Cochrane literature quality evaluation.

**Figure 4:**
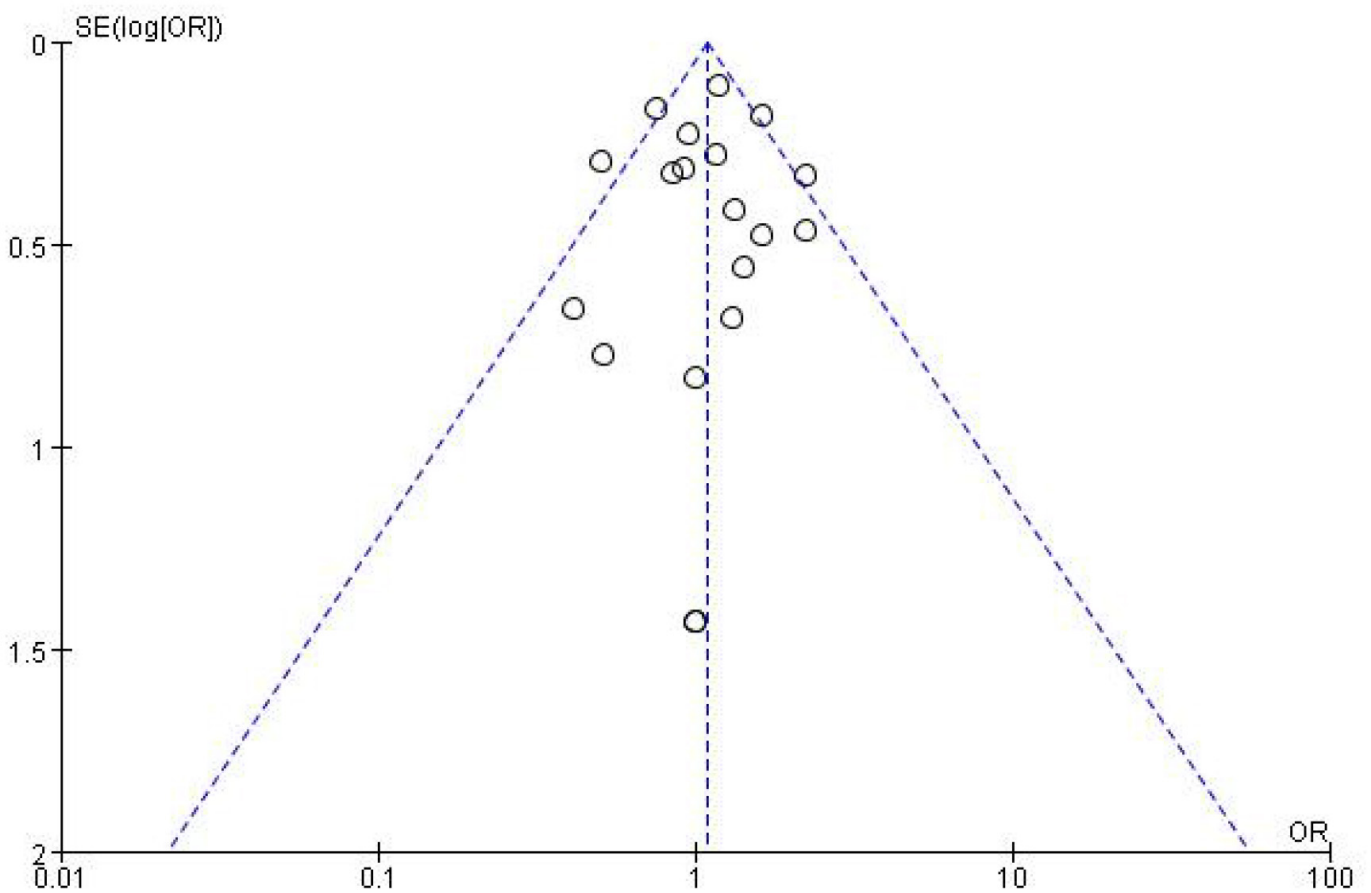
Funnel plot assessing publication bias for studies comparing metformin use and SGA incidence in the overall unclassified population.

**Figure 5:**
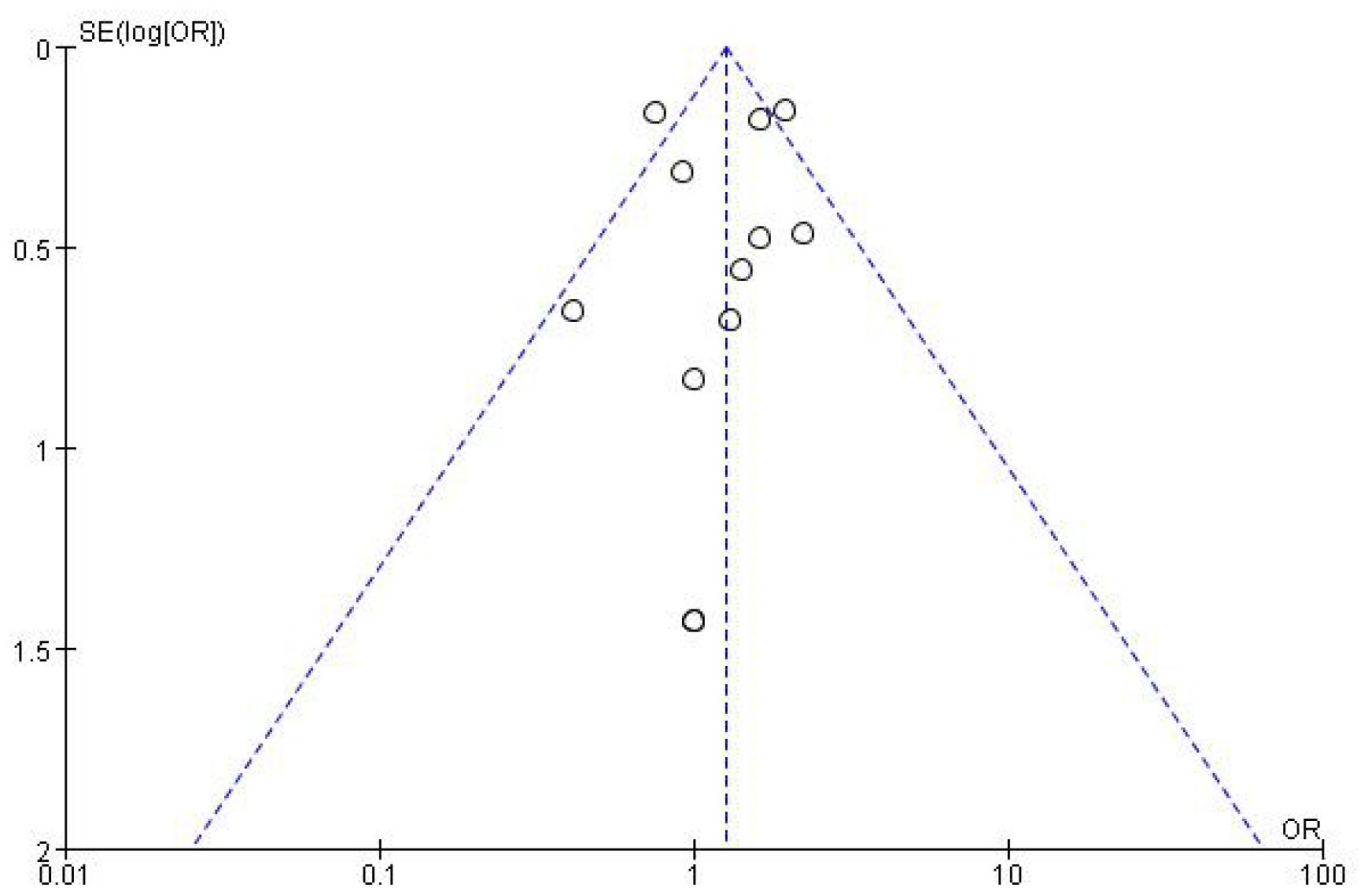
Funnel plot assessing publication bias for studies comparing metformin use and SGA incidence specifically in studies using insulin as comparator.

Risk of bias assessments reporting overall quality of included studies are presented in Figure 3. RCTs' risk of bias was evaluated collaboratively using the Cochrane Collaboration tool, which assessed seven domains (selection, performance, detection, attrition, reporting, and other biases) for each study and rated as low, unclear, or high risk (Figure 3). For each comparison, the level of certainty of the evidence ranged from low to high. The Newcastle-Ottawa Scale (NOS), which evaluates eight domains of bias, was applied to assess observational studies. The median Newcastle-Ottawa score for the 19 studies reviewed was 7, with quality scores ranging from 6 to 8 for cohort studies and consistently at 7 for case-control studies. Based on these evaluations, all studies were considered high quality (Table 1). The primary outcome measure was the unadjusted odds ratio (OR) for binary outcomes. Meta-analysis was conducted using Review Manager (RevMan) version 5.4. Funnel plots were generated to assess publication bias in datasets with more than nine trials comparing the metformin group to the unclassified population or the insulin group (Figures 4, 5). Heterogeneity was quantified using the *I*^2^ statistic to identify variability due to non-random factors.

**Table 1:** The bias of the observational studies.

**References**	**Selection**				**Comparability Control for important factor**	**Exposure**			**Scores**
	**Adequate definition of cases**	**Representativeness of the cases**	**Selection of controls**	**Definition of controls**		**Ascertainment of exposure**	**Same method of ascertainment for cases and controls**	**Nonresponse rate**	
Brzozowska et al. (28)	★	★	★	★	★✩	★	★	★	6
Brand et al. (26)	★	★	★	★	★★	★	★	★	8
Tertti et al. (12)	★	✩	★	★	★✩	✩	✩	★	7
Balani et al. (13)	★	✩	★	★	★✩	✩	★	★	7
McGrath et al. (19)	★	✩	★	★	★✩	★	★	★	7
Fornes et al. (27)	★	★	★	★	★✩	★	★	★	8
Bowker et al. (17)	★	★	★	★	★✩	★	✩	✩	6
Lin et al. (22)	★	★	★	★	★★	★	✩	★	8
Yu et al. (30)	★	★	★	★	★★	★	✩	★	8

The Newcastle-Ottawa Scale (NOS) was used to assess the bias of the observational studies. All of which included in this analysis were rated as having a low risk of bias. Studies with a score of 6 or more were classified as high quality and included. studies with < 6 points in the screening process were directly excluded, and the scores are no longer given in detail here, which corresponds to “not high quality cohort studies” in the flow chart.

## Results

### Study and quality characteristics

Our search identified 1,069 records. After removing duplicates and screening titles, abstracts, and full texts, 19 studies involving 115,192 pregnancies were included (Figure 1). These studies included 19 couples of the experimental and control group. Of all the included studies, 50% were randomized controlled trials (RCTs). Table 2 provides an overview of the study designs and outcomes of the included studies. Notably, approximately 45% of the studies did not report data on race or ethnicity, which could potentially limit the generalizability of the results.

**Table 2:** Classification the included articles.

**Type of study**	**Metformin vs. insulin**	**Metformin vs. diet**	**Metformin vs. placebo**
Randomized controlled trials	6	0	3
Observational studies	6	2	0
Total	12	2	3

A detailed list of outcomes provided in the full report. The included articles were classified in detail according to their content to facilitate readers' subsequent analysis.

### Neonatal outcomes

**Figure 6:**
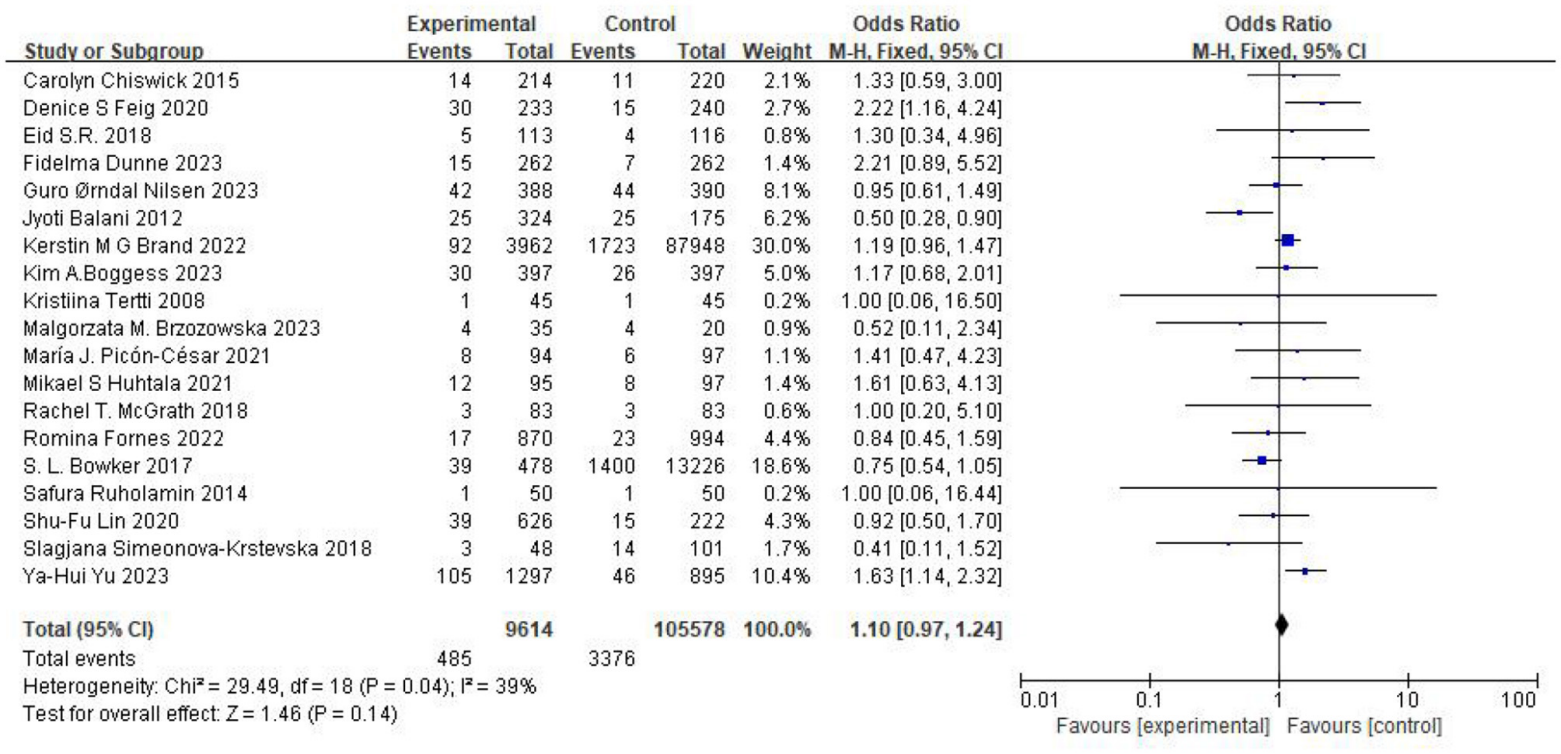
Studies evaluating the risk of SGA comparing metformin vs. unclassified population.

**Figure 7:**
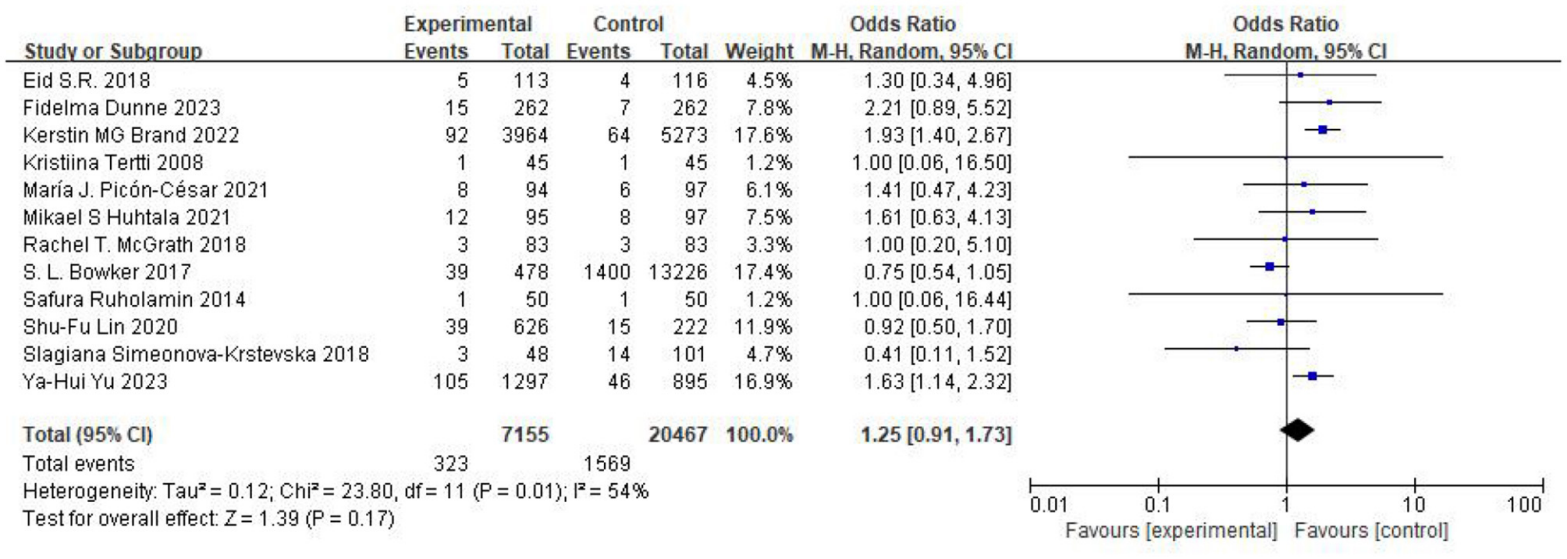
Studies evaluating the risk of SGA comparing metformin vs. insulin.

**Figure 8:**
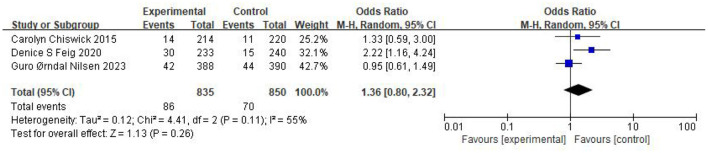
Studies evaluating the risk of SGA comparing metformin vs. placebo.

**Figure 9:**
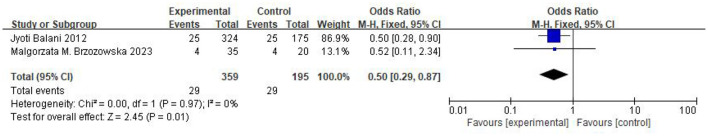
Studies evaluating the risk of SGA comparing metformin vs. diet modification.

The association between maternal metformin use and the risk of SGA was evaluated across four comparator groups. The pooled analysis results are summarized in Table 3: No significant difference: Metformin vs. Unclassified Population (*n* = 115,192) (OR 1.10, 95% CI 0.97–1.24, *p* = 0.14, *I*^2^ = 39%) (Figure 6) (12–31), Metformin vs. Insulin (*n* = 27,622) (OR 1.25, 95% CI 0.91–1.73, *I*^2^ = 54%) (Figure 7) (12, 14, 17–20, 22–25, 29, 30), Metformin vs. Placebo (*n* = 1,685) (OR 1.36, 95% CI 0.80–2.32, *I*^2^ = 55%) (Figure 8) (16, 21, 31). Metformin vs. Diet Modification (*n* = 554): The risk of SGA was significantly lower in the metformin group (OR 0.50, 95% CI 0.29–0.87, *I*^2^ = 0%) (Figure 9) (13, 28).

**Table 3:** Meta-analysis of SGA risk: metformin vs. different comparators.

**Comparator group**	**Number of participants (*n*)**	**Number of studies**	**Odds ratio (OR)**	**95% Confidence interval**	***I*^2^ statistic**	***p*-value**
Unclassified population	115,192	19	1.10	0.97–1.24	39%	0.14
Insulin	27,622	10	1.25	0.91–1.73	54%	0.17
Placebo	1,685	3	1.36	0.80–2.32	55%	0.26
Diet modification	554	2	0.50	0.29–0.87	0%	0.01

Compared with the first three groups, the metformin group demonstrated no significant difference in the risk of SGA. However, compared with the diet group, the SGA risk was significantly lower in the metformin group.

## Discussion

This meta-analysis, encompassing data from 115,192 pregnancies across 19 studies, provides the most comprehensive evidence to date regarding the association between metformin use for GDM and the risk of SGA. The principal finding is that metformin does not significantly increase the risk of SGA compared to insulin, placebo, or an unclassified treatment population.

The comparable SGA risk between metformin and insulin aligns with a previous meta-analysis, reinforcing the reliability of this finding (32). Although some individual studies mentioned a potential increased risk of SGA with maternal metformin use, our statistical analysis did not support this concern. The reason might be that metformin improves maternal insulin sensitivity without substantially impairing placental function, thus exerting minimal adverse effects on fetal growth. However, as metformin crosses the placenta, the long-term effects on fetal growth and metabolism remain unclear, necessitating further study (8, 9).

A noteworthy finding is the significantly lower risk of SGA observed with metformin compared to diet modification alone (OR 0.50). This contrasts with the null findings in other comparisons and has not been highlighted in prior reviews.

This finding highlights the potential limitations of dietary interventions in achieving optimal glycemic control during pregnancy. This conclusion is based on limited data, as studies comparing metformin with diet are few and methodologically heterogeneous, warranting cautious interpretation (13, 28).

By incorporating a larger number of studies than previous reviews, this analysis provides a more comprehensive assessment of the association between metformin use and SGA risk. In the process of searching, we used the keywords with metformin, SGA, GDM and the synonymous substitutions, to assure more and complete articles being included.

A major strength of this study was the comprehensive analysis of multiple comparative groups. No previous meta-analysis has specifically compared metformin with multiple alternative treatments regarding SGA incidence, highlighting the unique contribution of our study. Conversely, insulin therapy should remain the preferred option in high-risk populations, including those with pre-existing fetal growth concerns or contraindications to metformin use.

However, there were several limitations. Variations in GDM diagnostic criteria, treatment protocols, metformin dosing (ranging from 500 mg to 2500 mg daily), patient baseline characteristics (e.g., age, BMI, ethnicity—with approximately 45% of studies not reporting ethnicity) contributed to the moderate-to-high statistical heterogeneity observed (*I*^2^ up to 55%) (9, 33) (Table 4). It suggests that the overall “no significant difference” finding may mask variable effects in different patient subgroups or clinical settings. Additionally, the complete absence of long-term follow-up data in the included studies represents a major evidence gap. While our analysis focused on neonatal SGA, it provides no information on potential long-term developmental, metabolic, or cardiovascular outcomes in children exposed to metformin *in utero*. Therefore, the absence of an overall significant association should not be interpreted as uniform safety across all clinical contexts, and individualized treatment decisions remain essential. This limitation precludes a comprehensive safety assessment and means that current clinical recommendations based on short-term neonatal outcomes must be made with caution, acknowledging this unknown. In clinical practice, this means that while metformin may be appropriate for many women with GDM, insulin may remain preferable in pregnancies with existing concerns about fetal growth or in populations not well represented in current studies. The limitations weaken the robustness and generalizability of these specific comparisons, and prevents a comprehensive evaluation of the potential developmental and metabolic effects of prenatal metformin exposure on offspring, preventing a definitive evaluation of metformin's long-term safety profile.

**Table 4:** The table summarizing study characteristics and sources of heterogeneity.

**No**	**References**	**Study design**	***N***	**Distribution**	**Age mean ±SD/range (y)**	**BMI mean ±SD/median (IQR) (kg/m^2^)**	**2 h OGTT mean ±SD/range (mmol/L)**
1	Chiswick et al. (16)	Randomized controlled trial	434	UK	28.7 ± 5.8	37.8 ± 4.9	NR
2	Feig et al. (21)	Randomized controlled trial	473	Australia, Canada	NR	NR	NR
3	Eid et al.(18)	Randomized controlled trial	229	NR	31.6 ± 3.6	29.44 ± 4.53	>8.6
4	Dunne et al. (29)	Randomized controlled trial	524	Ireland	34.3 ± 4.9	29.9 (25.6–33.7)	≥8.5
5	Nilsen et al. (31)	Randomized controlled trial	778	NR	29.7 ± 4.5	28.9 ± 6.4	NR
6	Balani et al. (13)	Case-control study	499	Asia, Africa	33.4 ± 5.4	30.2 ± 7.1	>7.8
7	Brand et al. (25)	Case-control study	91910	Finland	18–45	NR	≥8.6
8	Kim A.Boggess (15)	Randomized controlled trial	794	USA	18–45	36.4 ± 8.0	NR
9	Tertti et al. (12)	Case-control study	90	South Western Finland	32.7 ± 4.8	33.2 ± 6.2	8.9 ± 2.0
10	Brzozowska et al. (28)	Cohort study	55	Asia	31.9 ± 4.9	25.8 ± 4.7	NR
11	Picón-César et al. (24)	Randomized controlled trial	191	Spain	18–45	NR	NR
12	Huhtala et al. (23)	Randomized controlled trial	192	Finland	30.3 ± 5.2	29.1 ± 5.5	≥8.7
13	McGrath et al. (19)	Case-control study	166	Australia	33.1 ± 4.8	27.8 ± 8.0	>8.5
14	Fornes et al. (27)	Cohort study	1864	Swedish	32.4 ± 5.0	26.9 ± 4.5	NR
15	Bowker et al. (17)	Cohort study	13704	Canada	30.5 ± 3.3	25.4 ± 2.0	NR
16	Ruholamin et al. (14)	Randomized controlled trial	100	USA	24.6 ± 6.3	26.4 ± 2.8	NR
17	Lin et al. (22)	Retrospective cohort study	848	China	34.05	NR	NR
18	Simeonova-Krstevska et al. (20)	Randomized controlled trial	149	NR	32.2 ± 4.7	28.8 ± 5.3	< 8.5
19	Yu et al. (30)	Cohort study	2192	UK	18–45	NR	NR

BMI, body mass index; SD, standard deviation; NR, not record; OGTT, oral glucose tolerance test.

To address the limitations of current evidence and translate these findings into clearer clinical guidance, future research should prioritize the following: Standardization of Study Protocols: prospective studies, especially randomized controlled trials, should adopt consensus-based, standardized protocols for GDM diagnosis, metformin dosing, and comparator treatments. This will reduce methodological heterogeneity and facilitate more robust and comparable meta-analyses in the future. Long-term Offspring Follow-up: the focus of research must extend beyond birth outcomes. Large-scale, longitudinal cohort studies are imperative to evaluate the long-term cardiometabolic health, neurodevelopment, and growth trajectories of children exposed to metformin *in utero*. Such data are essential for a comprehensive risk-benefit assessment of metformin use in pregnancy.

Overall, maternal oral metformin can indeed reduce newborn weight to a certain extent (metformin can prevent LGA to a certain extent), but the data tend to presents a milder effect that has not reached the level of SGA. The potential for its broader clinical application remains promising. Some studies provided proof of concept that treatment of preterm pre-eclampsia and pregnancy-related hypertension is possible (34, 35). Mechanistic studies exploring how metformin influences placental function and fetal development may further elucidate its safety profile. Through this analysis, we currently agree on the safety of metformin, it is reasonable to use metformin in those with type 2 diabetes and chronic hypertension or nephropathy in pregnancy (36).

## Conclusion

This meta-analysis suggests that metformin is a cost-effective and generally safe treatment option for GDM, with no significant association with increased SGA risk in the general population. Metformin presents a practical alternative for patients who cannot tolerate insulin or face significant financial constraints. Its affordability and ease of use make it an attractive option for low-risk pregnancies or resource-limited settings. Despite these promising findings, the need for further research remains critical to fully understand the long-term effects of metformin and to optimize GDM management strategies. A patient-centered approach that considers individual risk factors, treatment preferences, and socioeconomic circumstances is crucial for tailoring GDM management strategies (6, 8, 9, 36, 37).

## Data Availability

The original contributions presented in the study are included in the article/supplementary material, further inquiries can be directed to the corresponding authors.
